# Fine-tuned deep learning models for early detection and classification of kidney conditions in CT imaging

**DOI:** 10.1038/s41598-025-94905-2

**Published:** 2025-03-28

**Authors:** Amit Pimpalkar, Dilip Kumar Jang Bahadur Saini, Nilesh Shelke, Arun Balodi, Gauri Rapate, Manoj Tolani

**Affiliations:** 1 School of Computer Science and Engineering, Ramdeobaba College of Engineering and Management, Ramdeobaba University, Nagpur, Maharashtra India; 2 Department of Computer Science and Engineering (Cyber Security), School of Engineering, Dayananda Sagar University, Bangalore, India; 3 Symbiosis Institute of Technology, Nagpur Campus, Symbiosis International (Deemed University), Pune, India; 4 Department of Electronics and Communication Engineering, Dayananda Sagar University, Bengaluru, Karnataka India; 5 PES University, Bangalore, Karnataka India; 6 Department of Information and Communication Technology, Manipal Institute of Technology, Manipal Academy of Higher Education, Manipal, 576104 India

**Keywords:** Kidney disease classification, Computed tomography scans, Transfer learning, Hyperparameter-fine-tuning, Otsu’s binarization, Watershed segmentation, Distance transform, Biomedical engineering, Information technology, Computer science

## Abstract

The kidney plays a vital role in maintaining homeostasis, but lifestyle factors and diseases can lead to kidney failures. Early detection of kidney disease is crucial for effective intervention, often challenging due to unnoticeable symptoms in the initial stages. Computed tomography (CT) imaging aids specialists in detecting various kidney conditions. The research focuses on classifying CT images of cysts, normal states, stones, and tumors using a hyperparameter fine-tuned approach with convolutional neural networks (CNNs), VGG16, ResNet50, CNNAlexnet, and InceptionV3 transfer learning models. It introduces an innovative methodology that integrates finely tuned transfer learning, advanced image processing, and hyperparameter optimization to enhance the accuracy of kidney tumor classification. By applying these sophisticated techniques, the study aims to significantly improve diagnostic precision and reliability in identifying various kidney conditions, ultimately contributing to better patient outcomes in medical imaging. The methodology implements image-processing techniques to enhance classification accuracy. Feature maps are derived through data normalization and augmentation (zoom, rotation, shear, brightness adjustment, horizontal/vertical flip). Watershed segmentation and Otsu’s binarization thresholding further refine the feature maps, which are optimized and combined using the relief method. Wide neural network classifiers are employed, achieving the highest accuracy of 99.96% across models. This performance positions the proposed approach as a high-performance solution for automatic and accurate kidney CT image classification, significantly advancing medical imaging and diagnostics. The research addresses the pressing need for early kidney disease detection using an innovative methodology, highlighting the proposed approach’s capability to enhance medical imaging and diagnostic capabilities.

## Introduction

Around the world, kidney stones are a public health concern. It is a condition where things in the urine crystallize and start forming into stones, creating severe pain and urinary issues. It is a significant challenge to prevent stone recurrence, especially in patients with a previous history of kidney stones, since stone recurrence in these patients is associated with an increased risk of renal failure. Blood tests and urine collections are part of metabolic evaluations that give a person a risk profile. The most familiar calcium stones result from excess calcium excreted in the urine, while genetic conditions can cause cystine stones. The provision of personalized preventive strategies based on metabolic evaluations is vital. More effective prevention and treatment are the goal of ongoing research into innovative therapies and precision medicine. A metabolic evaluation is necessary to avoid kidney stone recurrence to offer practical prevention direction. Initial crystals are microscopic and can grow into stones in weeks or months. High urinary calcium, certain diets, and low fluid intake are contributing factors. Kidney stones, also known as solid deposits, block urine flow and may harm health. Complications can be prevented when they are detected as quickly as possible. The recurrence of kidney stones becomes a formidable challenge in healthcare. Prone to stone formation: a metabolic evaluation, Alelign and Petros outline in 2018^1^, relies on a simple blood test and 24-h urine collection. This detailed assessment allows us to understand the individual’s metabolic profile and, from that, provide targeted prevention strategies that can be given to the patient to observe.

Since different stones require different prevention strategies, it is critical to understand what type of kidney stone is forming. Urine and stone composition analysis is crucial in identifying the leading causes and guiding the appropriate measures to prevent a recurrence. By conducting these tests, you can tailor strategies to individual patients and better manage the risk associated with kidney stones. Proper diagnosis and treatment of kidney stones require that they are detected and classified. Ultrasound, X-ray, and CT scanning are traditional methods of diagnosing kidney stones. Traditional detection methods such as ultrasound and X-ray are tremendous but rarely deliver sufficient diagnostic accuracy. Such limitations are addressed by the study^2^ that applies advanced computer-aided detection techniques that increase diagnostic accuracy while harnessing the potential of CT data effectively. Using kidney stones detection and classification of kidney tumors has become very popular in medical research using deep learning. With deep learning, computer algorithms perform advanced medical image analysis and diagnose accurately. For example, we have deep learning models to help detect kidney stones on CT scans. These models have been proven to accurately identify and classify kidney stones, a crucial technology in correctly diagnosing and treating kidney stones. We also use deep learning models such as CNNs to analyze CT images and predict if the patient has kidney stones. Using deep learning algorithms, physicians can quickly identify kidney stones with high efficiency and undergo timely medical treatment to prevent possible risks^3^.

Moreover, transfer learning and ensemble methods have substantially enhanced kidney stone detection models^4^. Transfer learning involves using the knowledge gained from one task to solve a different task, and ensemble methods attempt to outperform a single approach by combining multiple ones. These techniques produce highly accurate systems that allow medical professionals the time to diagnose defective goods inexpensively. Integrating these advanced methodologies has transformed kidney stone detection, solving the bottlenecks in the current diagnosis methods. With an increase in kidney-related health issues, such innovative approaches are necessary to identify and rapidly identify patients and reduce the complications that would arise from having kidney stones. Known as a significant global public health problem, kidney stones can cause significant pain and, if undetected, lead to complications. However, traditional diagnostic methods, such as ultrasound and X-ray, do not have high accuracy and sensitivity; therefore, investigating more reliable diagnostic techniques is essential. The incidence of kidney-related issues is increasing, and lifestyle factors, along with other diseases like diabetes, make it necessary to identify and classify kidney stones promptly through new approaches.

The complex kidney structures, variability of stone formation, and the lack of adequate segmentation techniques in medical images make the problem of kidney disease detection and classification a critical challenge. Current diagnostic approaches often fail to provide clear and consistent segmentation for the tumor and leave the diagnosis subjectively sensitive to human perception. As a result, delays in diagnosis and suboptimal treatment planning often occur. Despite the success of medical image analysis using deep learning-based models, these models lack the robustness needed for kidney disease detection. Additionally, the existing literature recognizes two significant gaps: (1) a lack of well-developed precise segmentation methods that cannot clearly distinguish kidney stones from surrounding tissues and (2) ineffective optimization of deep learning models to classify kidney diseases. This study combines advanced image processing and deep learning techniques to address these challenges.

### Contributions and novelties of the paper

Based on this, we introduce an innovative technique for detecting and classifying kidney disease in routine CT images. It uses several advanced techniques (such as image processing, data normalization, and many augmentation capabilities). Key contributions include:

i. Watershed Segmentation yields a more accurate and detailed delineation of kidney disease than existing detection methods.
ii. An improvement of image clarity and effective segmentation of stones from surrounding tissues using Otsu’s Binarization Thresholding is applied to the image.
iii. Additionally, the research used the transfer learning capability of pre-trained models (VGG16, ResNet50, CNNAlexnet, and InceptionV3) to make transferable knowledge work toward the optimization of solving kidney disease classification problems.
iv. Normalizing the data and applying data augmentation techniques (zoom, rotation, shear, brightness adjustment, horizontal/vertical flip) were performed to ensure data uniformity and diversify training data, strengthening model robustness.
v. The approach was thoroughly assessed against different baselines and state-of-the-art methodologies to validate its effectiveness and efficiency.

Watershed segmentation is used to refine kidney structure delineation, which outperforms the accuracy of standard methods. Otsu’s binarization thresholding performs even better in improving image clarity by separating the stones from adjacent tissues. The research uses transfer learning to utilize pre-trained deep learning models (VGG16, Resnet50, CNNAlexnet, and InceptionV3), which best extract features for classification. Data normalization and augmentation help make the dataset uniform and robust; otherwise, the risk of overfitting might happen. Finally, a state-of-the-art comparative evaluation is provided to validate the effectiveness of the proposed approach. By filling these gaps, this study helps improve diagnostic accuracy, segmentation efficiency, and classification accuracy, thereby enhancing kidney disease detection and treatment planning. This approach provides a significant step toward reliable renal disease detection and classification, which can contribute to a nuanced understanding of renal health. The novelty is the combination of watershed segmentation and Otsu’s binarization thresholding to perform Otsu’s binarization and improve feature extraction from CT images, significantly increasing classification accuracy. Our approach uses data normalization and diverse augmentation techniques to achieve robust model performance over the dataset. Favorably, finely tuned transfer learning models, coupled with the processing of images and hyperparameter optimization, raised the accuracy of kidney disease classification to address the urgent need for early detection in clinical settings.

## Literature review

Artificial intelligence and machine learning have seen considerable progress in detecting and managing kidney stones in recent years. Deep learning has been proven invaluable in medical applications, as it can detect and localize kidney stones in CT images automatically. The resulting technique’s high accuracy and efficiency improve diagnostics and reduce the possibility of human error. Further, transfer learning has also emerged as a viable method to reuse a trained model for a new task. Through transfer learning, deep learning model performance is optimized in kidney stone segmentation by leveraging the knowledge of similar tasks, thereby providing a time-efficient and resource-saving approach toward model development. Implementing this method can address issues of adapting 3D convolution networks to clinical data collected from various modalities to which it has not previously been exposed^5^. Being the beneficial addition of AI and ML techniques in detecting and managing kidney stones, they are influential in improving diagnostic accuracy, lessening human errors, and giving personalized therapeutic approaches. Furthermore, these techniques would also help predict the therapy’s success rate and outcome and reduce the dose as low-dose CT techniques become available. Nevertheless, these limiting factors are still considered, including the lack of consideration for stone composition and the impact of AI algorithms on predicting the predicted rate of success and treatment outcomes.

### Segmentation methods

Accurately detecting and classifying kidney stones and other renal conditions from medical images requires segmentation. In recent research, deep learning-based segmentation techniques, along with other methodologies, have shown an increase in the performance of the segmentation. The orientation of this section was the most relevant study to our work, and it provided a solid reason for why they were included. One such study by Leube et al.^6^ demonstrates the usefulness of combining positron emission tomography (PET) with CT in kidney segmentation is one. Our hybrid approach improved segmentation accuracy, especially for kidneys with cysts or near adjacent organs. Adding PET data to the set of modalities enabled better delineation, demonstrating the utility of multi-modality imaging in renal diagnostics. In another study relevant to this, Junyu et al.^7^ used a semi-supervised deep learning method based upon cycle generative adversarial network (CycleGAN) for segmenting kidneys in magnetic resonance imaging (MRI). An approach yielded excellent results with a mean Dice score of 0.92 and a mean Jaccard score of 0.85 over 80 datasets. The techniques derived from MRI were shown to apply to CT images, demonstrating the flexibility of deep learning in segmentation tasks. In Gaikar et al.^8^, kidney segmentation was investigated using different MRI sequences with fivefold cross-validation on eight deep-learning model datasets. Their findings on T1-weighted non-Gd images emphasized that U-Net provided the most accurate segmentation with a dice similarity coefficient (DSC) of 89.34. A critical point was made by the study, which is that good segmentation accuracy relies substantially on model selection and tuning.

Felix et al.^9^ also exploited pixel-based identification techniques to detect kidney stone boundaries. Using their advanced segmentation method, they achieved an accuracy of 92.5%, showing that identifying precise boundaries between the stone and the kidney improves the clinical outcomes of patients with kidney stones. In a related study, Angshuman et al.^10^ found kidney stones in hospital and clinical ultrasound images. Their feature extraction method, a 3D U-Net model, obtained 96.82% accuracy. The research suggests that ultrasound imaging and advanced segmentation can improve kidney stone detection. The work of Li et al.^11^ extends the feasibility of deep semantic segmentation models in kidney segmentation and stone detection. These findings support the idea that deep learning can improve kidney-related diagnostics accuracy. Amiri et al.^12^ show how several image features, including renal radiation dose, irradiated renal volume, and 24-h urine volume, predict chronic kidney disease (CKD). They surmounted the obstacles of sparse data and generated 140 radiomic features, achieving 94% accuracy with Random Forest. Ma et al.^13^ proposed a heterogeneous modified artificial neural network (HMANN) to perform early CKD detection and kidney segmentation within the internet of medical things context. On ultrasound images, HMANN consists of a support vector machine (SVM) and multilayer perceptron (MLP) and has extremely high efficiency with speedy computation, high sensitivity, specificity, and an area under curve score of 92.2 for kidney segmentation. These studies point to the potential of advanced techniques for predicting and diagnosing CKD. While advances in segmentation processes have been made, thorny issues remain, namely the ability to detect stones accurately and perform segmentation efficiently. Existing methods lead to long execution times and prominent memory usage and need optimization for practical clinical applications. This can be confirmed by the previous studies indicating the need for optimization. Addressing these limitations is crucial for improving the reliability and efficiency of kidney disease diagnostics.

### Classification methods

With machine learning and deep learning techniques, kidney diseases have been explicitly classified for kidney stone patients. The discussion of the most relevant studies that advance understanding of the most practical classification methods is focused on how these studies are relevant toward better diagnostic accuracy and potentially better patient outcomes. Pande and Agarwal^14^ are notable ones who have applied the concept to classify different renal diseases using a multi-classification methodology. The researchers identified kidney cysts, stones, and tumors with an accuracy rate of 82.52% using a pre-trained YOLOv8n-cls model. By using advanced deep learning models, the work shows that precise disease classification is possible, which is a starting ground for future research in that area.

Saif et al.^15^ combined CNNs and LSTM networks to predict CKD occurrence in a proposed ensemble model. Their model, however, showed marked improvements in classification performance and suggested that the combination of different architectures could improve the diagnostic capabilities of each model. While this is promising for population-based studies, it may lack the ability to give personalized patient care. Subedi et al.^16^ focused on differentiating between four significant categories in CT scan images: Cysts, normal states, tumors, and stones. They enhanced the capabilities of a modified Vision Transformer (ViT) model, achieving optimal performance with a training/testing split of 90:10. The ViT model shows that it can classify many different pathologies of medical imaging with equal veracity, and in doing so, presents a modern view of kidney classification. Ugur Kilic et al.^17^ present a CNN-based computer-aided diagnostic system to detect kidney stones in DUSX images automatically. Their study used a novel dataset comprising 630 DUSX images and combined the YOLOv4 model with the CBC technique for pre-processing, the dataset achieved a remarkable accuracy rate of 96.1%. The effectiveness of YOLOv4 in accurately detecting kidney stones highlights the importance of model selection for achieving high diagnostic performance, reinforcing the need to choose the right model to ensure optimal results.

Yao et al.^18^ showed deep and shallow combination features derived from 18F-FDG PET/CT scans to predict EGFR-sensitizing mutations in non-small cell lung cancer (NSCLC). The work of their fusion model improves the precise identification of genetic mutations for personalized treatment of cancer. According to Wang et al.^19^, the Neuroendoscopic Parafascicular Evacuation of Spontaneous Intracerebral Hemorrhage (NESICH) is an effective technique for minimally invasive hematoma removal. The multi-center study is promising preliminary outcomes in ICH management. In light of the work by Li et al.^20,21^, the screening methods for patients with and without the interfering medications for PA were investigated. Their findings point to the importance of maximizing PA diagnosis for the management of hypertension. Fan et al.^22^ showed that gut microbiota was compared in peritoneal dialysis patients with peritoneal fibrosis. Their work confirms a relationship between the microbiota composition and peritoneal function, which may cause dialysis-related problems. Xingguang et al.^23^ presented a robotic bronchoscope system for endovascular localization and biopsy on pulmonary lesions. This innovation improves accuracy and access to lung cancer diagnosis, a trend for robotics in medical procedures. Zhang et al.^24^ brought forth an image-guided para-cortical spinal tract approach for hematoma evacuation in spontaneous intracerebral hemorrhage (ICH) patients. Image-guided neurosurgical interventions can play an essential role in this method of neurosurgery as it minimizes brain damage and increases patient outcomes.

Kumar et al.^25^ developed a new deep-learning model using a fuzzy deep neural network to classify and predict kidney disease. Their accuracy of 98.78% achieved with this model far surpassed existing methods. This shows that deep learning can further improve kidney disease classification when enwrapped by fuzzy logic. Bingol suggested a novel deep-learning model with convolution and batch normalization layers upon diagnosing kidney disease^26^. Although the model has a low layer count, it achieves high success rates, and the results highlight the significance of architectural design in building practical diagnostic tools. In a complementary work, Altalbe et al.^27^ developed a deep-learning CNN model for classifying kidney disease. The approach consisted of transferring digital imaging and communications in medicine (DICOM) images to jpg files, with the features extracted by a CNN giving back results that were better than those of existing methods. Together, these studies demonstrate the ongoing advances in deep learning models for improved kidney disease detection and classification, with improved accuracy and potential clinical applications.

Ashafuddula et al.^28^ developed an intelligent diagnostic system for early-stage renal disease using novel healthcare data. An elegant feature of this study was that data integrity was thus prioritized so that no loss could occur, even if some values were missing. To speed up model training and better perform, I use dimension reduction to reduce the space of features. The study looked at four datasets and identified adaptive boosting, logistic regression, and passive-aggressive classifiers for CKD analysis in real-life data. In forecasting unseen therapeutic CKD data, particularly early-stage cases, reliable CKD classification was achieved with 96.48% accuracy using an ensemble characteristics-based classifier.

Jerlin et al.^29^ proposed a new CKD classification method based on a fruit fly optimization algorithm for feature selection and multi-kernel SVM for classification. This study obtained a classification accuracy of 98.5% for the CKD dataset, which exceeds the results of the existing hybrid kernel-based SVM, fuzzy min–max GSO neural network, and SVM methods. Zhang et al.^28^ proposed an innovative approach for detecting kidney lesions in CT scans. They increased the visibility of small lesions using morphological cascade CNNs. Key components included a modified six-layer feature pyramid network for varying-sized feature maps and a four-layer cascade region-based CNN with high-precision detection. These cascade RCNNs were also validated in experiments, which confirmed their superiority over previous methods. However, the study shows how deep learning models can help automate the detection and classification of renal illnesses, helping to diagnose renal illness faster and more accurately, positively impacting patient outcomes. This collection of studies shows the advances in deep learning models for detecting and classifying kidney diseases. Researchers improve diagnostic accuracy and efficiency by using various architectures and methodologies. Then, we leverage these advances and integrate our work with transfer learning models, including VGG16, ResNet50, CNNAlexnet, and InceptionV3, to improve image classification of kidney conditions in CT images.

### Transfer learning methods

Malware detection using medical imaging as a motivating example, transfer learning has become a predominant strategy in medical imaging, allowing new tasks to reuse pre-trained models. This approach learns from neighboring tasks and optimizes deep learning model performance, which leads to a less time-consuming and, as a byproduct, less resource-consuming downstream model development process. Some recent studies, such as that by Sassanarakkit et al.^5^, have shown the state of the art of transfer learning in overcoming some difficulties with using a 3D convolution network in being adapted to different modality data. In 2023, Muneer Majid presented a computer-assisted diagnosis machine learning and fine-tuned transfer learning-based system for kidney tumor detection on CT images. We used extensive experiments to evaluate different deep learning models, with the conventional fine-tuned ResNet 101 and DenseNet 121 being the primary focus. At the same time, Mahalakshmi^30^ used an ensemble method that combines the classification results of three DCNNs to reduce variance and bias using Majority voting. An overall accuracy of 98.49% was achieved using Hyperparameter tuning using Gorilla Troops Optimizer. Furthermore, Badawy et al.^31^ also presented a renal disease classification framework using CT and histopathological images of kidneys through a CNN and transfer learning-based approach with superior accuracy compared to other approaches for renal disease classification. Therefore, the study’s objective was also to improve the classification of kidney diseases for accurate diagnosis and treatment.

Anari et al.^32^ suggested an explainable attention-based breast tumor segmentation model using UNet, ResNet, DenseNet, and EfficientNet. The resulting segmentation method is more accurate and retains the interpretability required for clinical decision-making. EfficientUNetViT is further developed by Anari et al.^33^ with EfficientUNet and a pretrained Vision Transformer for breast tumor segmentation. Transformer-based feature extraction is used with this method for segmentation efficiency and is shown to offer improved medical imaging performance. Sarshar et al.^34^ improved brain MRI classification using VGG16 and ResNet50 and the Multi-Verse Optimization (MVO) method. Their feature selection optimizes classification performance,they show how metaheuristic techniques can help in medical imaging. Kia et al.^35,36^ present a fusion model for MRI-based brain tumor identification using VGG16, MobileNet, EfficientNet, AlexNet, and ResNet50. Using this approach, we can effectively integrate several architectures to improve classification accuracy, providing a robust means to detect tumors. In 2024, Kia^36^ introduced an attention-guided deep learning model in the multi-criteria decision analysis for managing customer loyalty. One of the aspects that the study covers is the use of AI to analyze customer behaviour and optimize retention strategies.

Dalia Alzu’bi et al.^37^ utilized deep learning methods to study kidney tumor injuries and classify kidney tumor types. The researchers used VGG16, ResNet50, and two 2D-CNN models modified versions for analyzing features extracted from the renal CT scans. It was also a significant contribution because it used a unique dataset from King Abdullah University Hospital composed of 8,400 images of 120 adult patients with suspected kidney masses who underwent CT scans. An 80% training and 20% testing division was used on the dataset. Overall, the outcomes highlight the superiority of the 3 proposed 2D-CNN models, with 60, 96 and 97 percent detect accuracies for VGG16, ResNet50, and 2D-CNN, respectively. In a separate investigation, Lee and Aqil^38^ also studied variations in renal glomerular tissue diseases, focal segmental, normal, and sclerotic. They used allied and multivariate models to develop a technique to improve model accuracy, and excellent results were achieved using a combined model, which achieved an accuracy of as much as up to 97%. However, the processing time presented in the study was longer than the general model, but it was alleviated by using high-performance computing resources.

In addition, Parakh et al.^39^ evaluated the diagnostic efficacy of a cascaded deep learning system on unenhanced CT images of urinary stones. The impact of pre-train with labeled CT image in transfer learning is evaluated in their work, and they show that the GrayNet pre-trained model can obtain 95.4% in detecting urinary tract stones amongst all other pre-trained U-Net models. Transfer learning with enriched datasets was the focus of the study, which suggested the possibility of improving CNNs’ performance and generalization across scanners. Finally, as shown, AI/ML (deep learning and transfer learning) techniques can be beneficial in detecting and managing kidney stones. This research was created to advance systems for the early and accurate detection of kidney-related diseases, which are important to achieve better patient outcomes and healthcare efficiency. Combining these techniques can improve diagnostic accuracy, help reduce human errors, and prescribe individualized therapeutic strategies. Further work is needed to overcome the current limitations and unlock these approaches’ potential for the clinical management of kidney stone disease. The study shows the need for a continuing research effort to improve the performance of these methods for practical implementation in healthcare applications. The studies above show that transfer learning works well for kidney disease classification, yet they also have some shortcomings. This could be because some of these models require great computational resources and cannot be applied practically in the clinical context. At the same time, the models rely on pre-trained models that need to be carefully scrutinized for the features learned from the source datasets to be viable for the target task.

### Recent advancements in kidney disease detection

With deep learning and transfer integration, hyperparameter optimization, kidney disease detection, and classification have been made much better. Recent studies have shown that these technologies improve diagnostic accuracy. For instance, Kumar et al.^25^ developed a fuzzy deep neural network model that predicted kidney diseases with high accuracy compared to other developed methods. Besides ensemble methods and hybrid models, results promise to improve classification accuracy. In their research^40^, Ashafuddula et al. show that an ensemble characteristics-based classifier is effective for CKD analysis and achieves an accuracy of 96.48% in early-stage cases.

Table 1 summarizes recent advancements in transfer learning and hyperparameter optimization, highlighting their contributions and limitations in kidney disease classification. Here, key developments like applying pre-trained models, fine-tuning techniques, and an ensemble learning approach are used to improve diagnostic accuracy. However, challenges like generalization problems, computational cost, and complexity remain in managing the model. However, ViT and CycleGANs have shown promise in additional methods, requiring large datasets and many computational resources. This comprehensive overview qualifies the evolving enigmatic nature of intelligence within medical imaging and dedicates further exploration, showcasing the imperative for further research to address current restricting factors and improve patient outcomes.

**Table 1:** Promising developments and persistent obstacles for kidney disease classification.

Advancement	Description	Limitations	References
Transfer learning techniques	They used pre-trained models (e.g., ResNet, VGG) to enhance the accuracy of kidney disease classification	It does not generalize well to all datasets; performance heavily depends on the source dataset quality	^2,5,41^
Vision transformer models	Use modified Vision Transformer (ViT) models to classify CT scan images effectively	It requires large datasets for practical training and may be less interpretable than traditional CNNs	^16,27^
Fine-Tuning Techniques	Fine-tuning pre-trained models to adapt them to specific tasks improves diagnostic performance	It requires careful selection of hyperparameters; it can lead to overfitting if not appropriately managed	^16,42^
Hyperparameter optimization	Advanced methods like Bayesian optimization and genetic algorithms to optimize model parameters	Computationally intensive; may require extensive resources and time for large datasets	^14,29^
Fruit fly optimization algorithm	They utilized optimization algorithms for feature selection in CKD classification	It may be sensitive to parameter settings and requires careful tuning to achieve optimal results	^14,29^
Hybrid models	The authors integrated different architectures (e.g., CNNs with LSTMs) for improved prediction capabilities	Complexity in training and tuning may require large amounts of labeled data for practical training	^15,28^
Ensemble learning approaches	They combined multiple models to enhance classification accuracy and robustness	Increased complexity in model management; potential for longer inference times	^25,40,43^
Deep semantic segmentation models	The authors utilized deep semantic segmentation for kidney segmentation and stone detection	Image noise can affect performance and may require extensive labeled data for training	^11,12^
Cycle-GAN for segmentation	They utilized Cycle-GAN for semi-supervised kidney segmentation, achieving high accuracy	Limited to specific imaging modalities; requires substantial computational resources for training	^6,7^
Segmentation and detection approaches	Enhanced kidney segmentation using deep learning techniques, improving the detection of lesions and stones	Limitations in detecting small lesions: segmentation accuracy can vary based on image quality	^7,9^
Multi-classification approaches	Use of multi-classification models to categorize various renal diseases effectively. The dual-augmentation method is used to achieve high accuracy	They may struggle with imbalanced datasets and require careful handling of class distribution in training	^14,42,20,21^
Automated CNN classification approaches	The researchers developed a CNN-based system for automated detection of kidney stones in ultrasound images	Performance may degrade with lower-quality images and limited generalization across different imaging modalities	^17,40^
Fuzzy deep neural networks	The researchers incorporated fuzzy logic in deep learning models for improved prediction of kidney diseases	Complexity in model design may require extensive tuning to achieve optimal performance	^25,26^
Morphological cascade CNNs	Innovative use of morphological cascade CNNs for enhanced detection of small kidney lesions	Complexity in architecture design may require extensive training data for effective learning	^29,28^

## Methodology

**Fig. 1:**
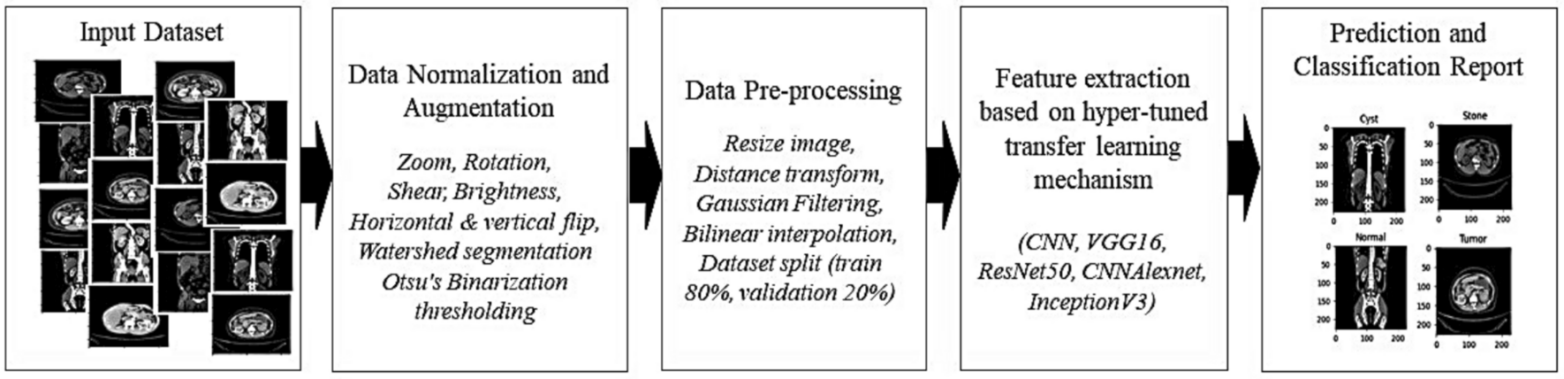
Proposed approach for kidney disease classification based on hyperparameter tuned transfer learning mechanism.

This section presents our advanced methodology for CT scan-based kidney disease detection. The details of pre-processing, data augmentation, and 4 different architectures for our kidney tumor diagnosis models are included. Our study pioneers a four-way multi-classification solution, employing a hyperparameter-tuned transfer learning mechanism for optimal model performance. The strategic use of CNNs facilitates the identification of intricate patterns in medical images, generating feature maps crucial for accurate diagnosis. As shown in Fig. 1, our approach combines cutting-edge technologies, ensuring superior sensitivity and specificity in kidney disease detection, marking a significant advancement in precision medicine for tailored treatment plans.

The study uses the CT Kidney disease dataset from Kaggle, which has been normalized and augmented (zoom, rotation, shear, brightness, flips) using ImageDataGenerator. Resizing images (227 × 227), applying a distance transform threshold (0.7), applying a Gaussian filter, and then bilinear interpolation for enhancement are some of the pre-processing done to images. Precise kidney disease detection is achieved for segmentation using watershed segmentation and Otsu’s binarization thresholding. Transfer learning models VGG16, ResNet50, CNNAlexNet, and InceptionV3 are used for feature extraction. It classifies the images into cyst, normal, stone, or tumor using ReLU, softmax activation, Adam optimizer, and Sparse Categorical Cross Entropy (SCCE) loss. Accuracy metrics are used to evaluate the performance. The results include training and validation loss, accuracy reports and a confusion matrix for robust performance assessment. These methods optimize segmentation and classification to enhance kidney disease detection diagnostic accuracy.

### Dataset

This research utilized a large, publicly available dataset to train a deep-learning model for kidney stone detection in CT images. The Kaggle dataset contains 12,446 kidney CT images neatly organized into four groups: healthy kidneys, those with fluid-filled sacs called cysts, those with abnormal growths known as tumors, and those with stone-like structures.

**Fig. 2:**
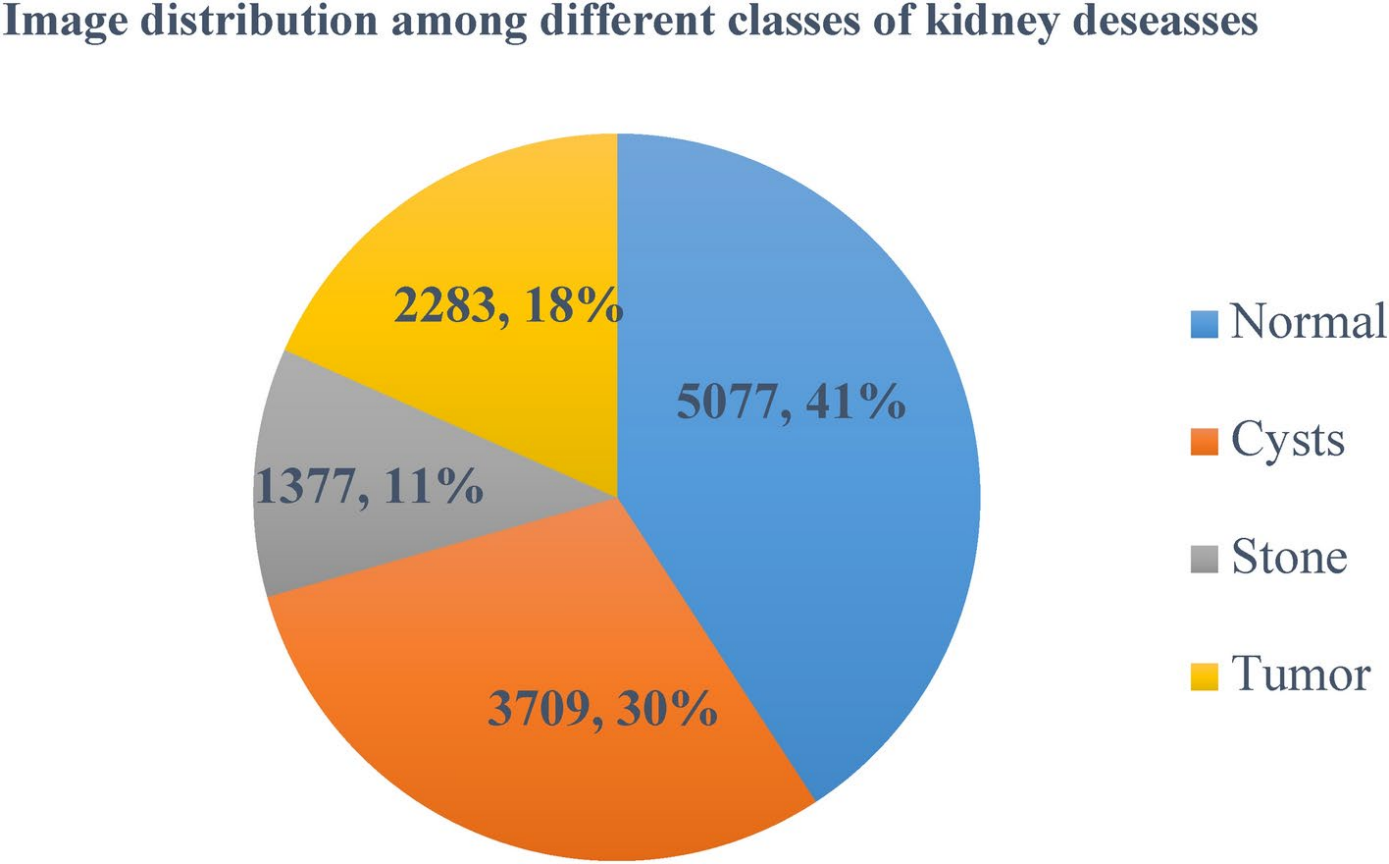
Dataset statistics based on different classes.

These images are valuable for researchers and scientists studying kidney disease^44^. These CT images are organized into various categories, along with their count, as illustrated in Fig. 2. The dataset was split: 80% for training (9,957 images) and 20% for validation (2,489 images). The model was trained on the training data to identify kidney stones in CT scans. Image pre-processing involves scaling and applying Gaussian filtering to remove noise, potentially improving diagnosis and treatment for kidney stone patients. By enhancing image quality, these techniques facilitate more accurate detection of kidney stones, ultimately leading to better patient outcomes.

### Data pre-processing and augmentation

In the past, researchers aimed to improve the accuracy of kidney disease diagnosis using deep-learning models trained on medical images. One key challenge they faced was the limited availability of such images. In our research, we employed data augmentation techniques to overcome the challenge of limited medical image availability for kidney disease diagnosis. Data augmentation techniques are used to expand the dataset and enlarge feature diversity to improve the model’s performance. It also helps the model to be more generalizable by dealing with variations in kidney CT images, like differences in scale, brightness, orientation, and anatomical structure. The models can be overfitted without augmentation, memorizing training data instead of learning meaningful patterns and performing poorly on instances it has never seen. Zoom, rotation, shear, and brightness adjustments are made using augmentation techniques to ensure that the model learns invariant features that aid its ability to recognize kidney disease, cysts, and tumors in the presence of variations in poses imaging conditions. Without augmentation, the model learns to be biased towards specific image characteristics and may not work well in real-world clinical diagnostic cases. Our dataset was first converted raw DICOM images to manageable JPG format and resized to a standard 227 × 227 pixels, ensuring uniformity and bilinear interpolation used during resizing to maintain image quality. Using the ImageDataGenerator function available with Keras, data augmentation created new images by zooming, rotating, and flipping existing ones, adding diversity to the training dataset. These newly generated images were created on the fly during training, saving storage space. The specific augmentation settings were carefully chosen and documented in Table 2. Augmentation was applied only to the training dataset, not the validation set. To address class imbalance, we utilized oversampling of minority classes and undersampling of majority classes to create a balanced dataset. We also employed weighted loss functions during training, penalizing misclassifications of minority classes more heavily. These strategies enhanced the model’s ability to generalize across all classes, improving the accuracy and reliability of kidney disease classification. Through the process of data augmentation, data prevents overfitting, reduces bias, and improves generalization, which is a critical factor in considering accurate and reliable kidney disease classification, an essential step in medical image analysis.

**Table 2:** Data augmentation parameter.

Augmentation parameter	Value
Pixels resizing	227 × 227 pixels
Interpolation	Bilinear
Segmentation	Watershed
Zoom, rescale	1./255
Rotation	15
Shear	0.05
Width_shift	0.05
Height_shift	0.05
Brightness_range	[0.1, 1.5]
Horizontal_flip	TRUE
Vertical_flip	TRUE
Thresholding	Otsu’s Binarization
Distance transform threshold	0.7
Filtering	Gaussian

The model was trained on the transformed training samples to learn more robust features, enhancing the diagnostic accuracy. Thus, we believed that a diverse, artificially enlarged dataset offered by this approach would allow the model to learn better and have better diagnostic accuracy for kidney disease. Research hoped that if the model were shown more examples of kidney images with varying degrees of similarity to one another, the model would be trained to learn the subtle signs of disease, even in unseen images. The research demonstrates the capability of data augmentation to combat the problem of low medical image data. By artificially expanding datasets, researchers can potentially improve the performance of the deep learning model used for medical diagnosis. In other words, the performance of deep learning can be increased artificially, which will help patient care. The research uses watershed segmentation to help detect kidney stones within the CT scan. However, this technique provides several benefits and significant advantages over other peer methods. Watershed segmentation is a robust image processing algorithm that can successfully keep apart touching or overlapping objects in an image. It can do as much as accurate identification of the boundaries of the stones, as well as precise segmentation and analysis in this context of kidney stone detection.

**Fig. 3:**
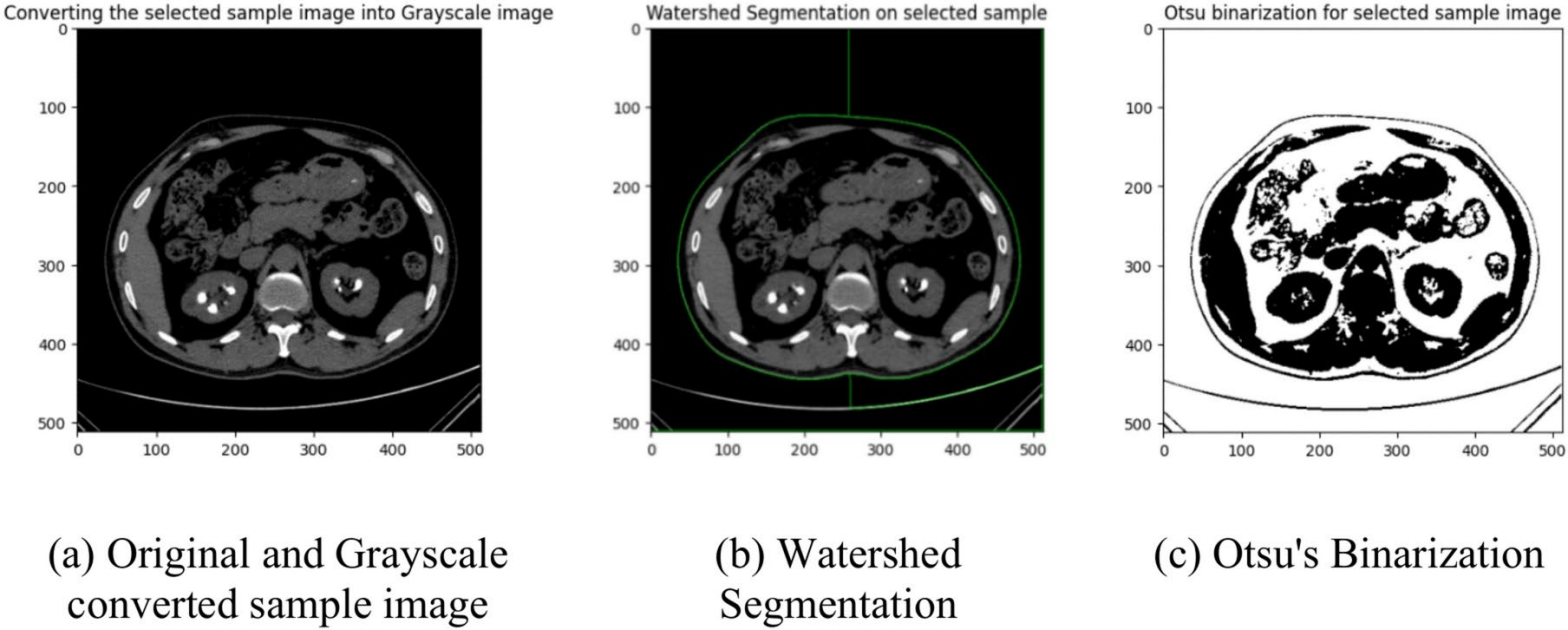
An illustration of applying image pre-processing steps.

Watershed segmentation performs better in recognizing and segmenting kidney stones in CT scans than in other methods, like threshold and edge-based. Watershed segmentation can help improve the accuracy and reliability of kidney stone detection and is essential for obtaining correct diagnosis and treatment. Otsu’s binarization is an image thresholding method that binarizes an image using pixel intensity values. The benefit of Otsu’s binarization over other peer methods is that it provides a more accurate threshold value by maximizing the between-class variance. These results are shown in Fig. 3, which shows a better separation of the image’s background and main objects. The significant role of watershed segmentation and Otsu’s binarization thresholding in refining feature maps for kidney CT image analysis is demonstrated as they significantly contribute towards better region delineation and enhancement of contrast for better classification. Effective in detecting the kidney structures, the watershed segmentation exploits the intensity gradient of an image as a topographic surface. It allows the approach to segment kidney regions, stones, cysts, and tumors based on boundaries, even with weak intensity variations. This help the deep learning model concentrate better on well-dedicated and sensible anatomical structures than squinty or confounding territories. An Otsu’s binarization thresholding helps segment the foreground kidney structures from the background, and to optimize the segmentation process, the best threshold is determined automatically. Compared to other methods, this method is handy for marking stone formations and cysts, in which pixel intensity variations can be subtle. Otsu’s method improves contrast, making minor, critical abnormalities distinct enhancing feature extraction. Combining these techniques improves feature map clarity, reduces spurious artefacts and enhances the model’s ability to learn highly structured patterns in kidney CT images, resulting in improved classification performance and higher diagnostic reliability.

**Fig. 4:**
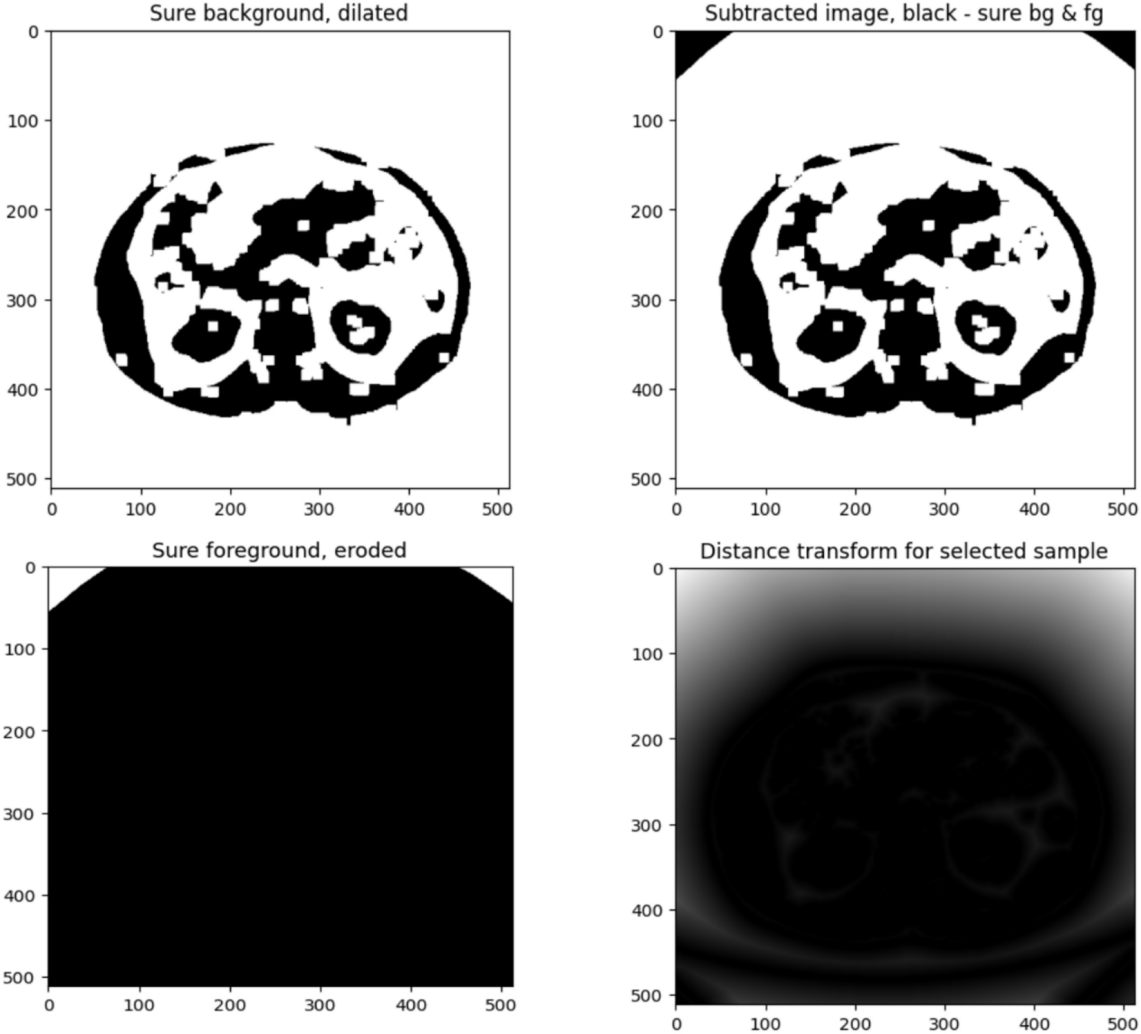
An illustration of applying foreground, background, and the subtracted information along with distance transformation on the sample image.

**Fig. 5:**
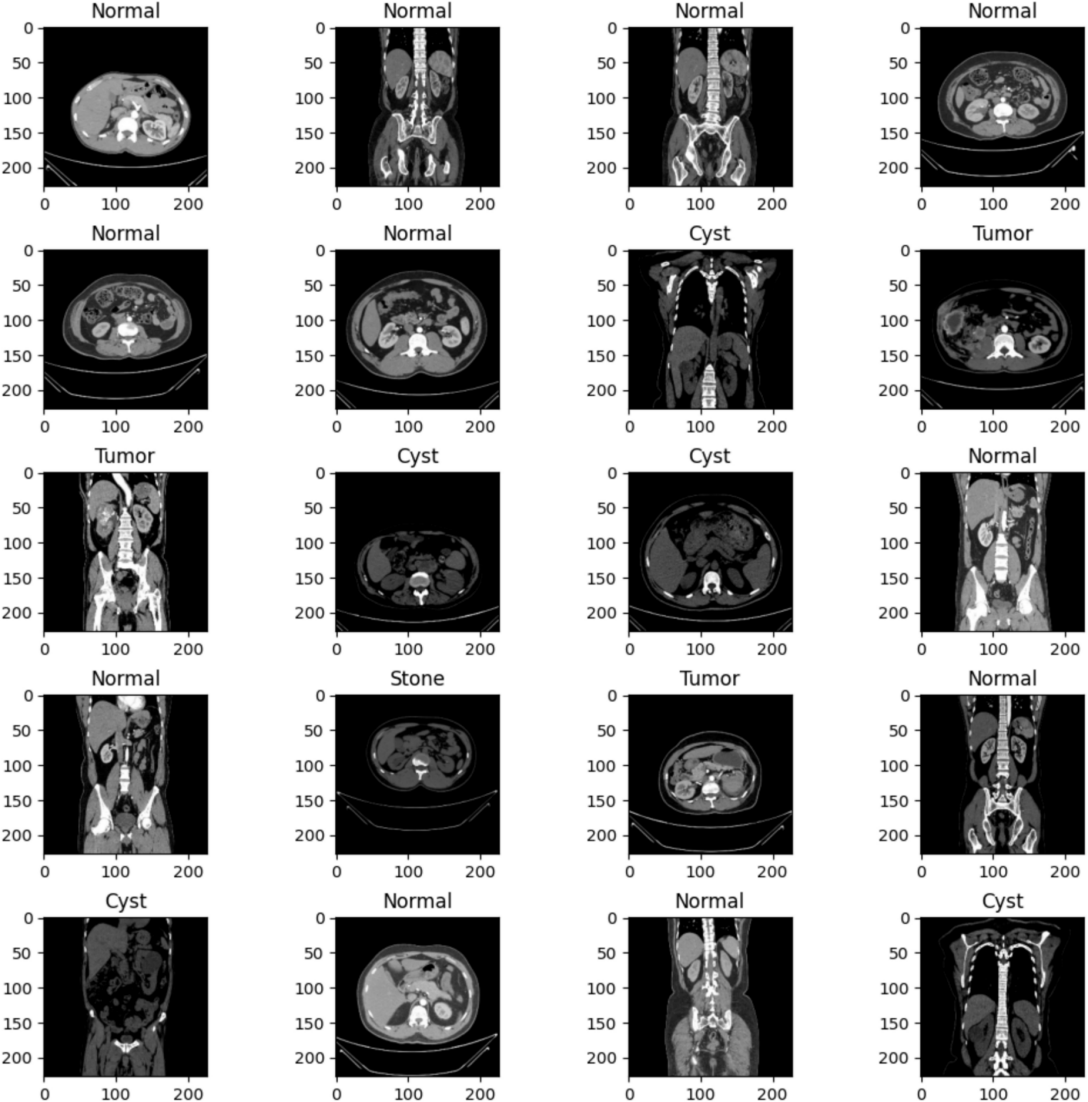
An illustration of sample dataset images with appropriate labels as in the dataset.

After applying Otsu’s binarization for noise removal, the next step in the proposed research was to perform a distance transform. As shown in Fig. 4, the background is denoted in black, and the second image is the distance transformed and thresholded. The white area represented the unknown area. The Distance transform is a mathematical operation that transforms each pixel’s value to its distance from the nearest boundary pixel. This operation is functional in image processing applications such as object recognition and segmentation. The transformation yielded a grey-level image resembling the input, where grey intensities within foreground regions denote distances to the nearest boundary pixel. Specifically, the distance transform replaced each pixel in a binary image with its proximity to the closest background pixel. This mapping assigned the distance of every pixel to the nearest obstacle, with the nearest usually a boundary pixel in binary images. This widely used method is essential for spatial analysis and has been heavily studied in the literature. Figure 5 shows a sample dataset image and its respective actual label. The pinpoint process of dataset labeling assigns tags or labels to raw data with images, videos, text, or audio. Since these labels denote the object class, they help model learning and pattern recognition. Even the time and cost invested required for dataset labeling are vital for creating outstanding models. This matches the understanding that meticulous labeling facilitates model efficiency, which is a role it plays in the reliable development of quality models.

### Hyperparameter optimization and tuning

Deep learning models require hyperparameter optimization to be run properly. It has been increasingly common to automate this process using recent methodologies such as Bayesian optimization and genetic algorithms. Exploring this hyperparameter space is more efficient using these techniques, resulting in better model accuracy and generalization. One example is an ensemble Pande and Agarwal^14^ method that combines hyperparameter optimization methods and can boost classification performance for multiple renal disorders. Advanced optimization strategies save computational costs and increase the models’ reliability to be used in clinical settings.

The transfer learning model performance for disease detection is improved by hyperparameter tuning, which is a key phase of improving the model’s performance. This changes different hyperparameters to make the model more accurate and efficient. As shown in Table 3, the following Ten key hyperparameters were modified to achieve this. Optimal parameters were chosen through experimentation with better outcomes. The selection process was underpinned by empirical evidence, and outcomes were fed back into the decision-making process. Our kidney disease classification system heavily relies on the performance of transfer learning models and the selection of hyperparameters. In particular, we chose four pre-trained models, VGG16, ResNet50, CNNAlexnet, and InceptionV3, since they are known as good performers in medical images and can deal with highly detailed CT-scanned data. Prior studies have shown that these models have been very accurate, making them a good fit for classifying kidney conditions accurately, which we want to achieve in this work.

**Table 3:** Hyperparameter tuned for the modified models.

Sr. no	Hyperparameter	CNN	VGG16	ResNet50	CNNAlexnet	InceptionV3
1.	Activation Layer	ReLu, Softmax	ReLu, Softmax	ReLu, Softmax	ReLu, Softmax	ReLu, Softmax
2.	Dense Layer	128	1024	1024	4096	1024
3.	Dropout	0.5	0.2	0.2	0.5	0.2
4.	Learning Rate	0.0001	0.0001	0.0001	0.0001	0.0001
5.	Batch Size	156	156	156	156	156
6.	Optimizer	Adam	Adam	Adam	Adam	Adam
7.	Pooling	MaxPooling2D	–	–	MaxPooling2D	–
8.	Epochs	25	25	25	25	25
9.	Loss	Sparse Categorical Cross Entropy	Sparse Categorical Cross Entropy	Sparse Categorical Cross Entropy	Sparse Categorical Cross Entropy	Sparse Categorical Cross Entropy
10.	Weights	–	Imagenet	Imagenet	–	Imagenet

In our methodology, we first selected hyperparameters like learning, batch size, and number of epochs based on existing practices and similar studies. We ran additional experiments with different hyperparameters employing random search and Bayesian optimization to improve our results. To do hyperparameter tuning, we used a grid search-based approach to systematically explore different combinations of learning rates, batch sizes, and epochs. By iterating the models’ training with different hyper parameter setting and measure its performance on a validation set, we could find the best setting to avoid overfitting and maximize the classification accuracy. Our models work best with a learning rate of 0.001, batch size of 156, and when learning on 25 epochs. Using this approach, we can analyze the effect of each parameter on the classification accuracy and overall model robustness, resulting in corroborating results that increase the reliability of the findings and allow us to understand better the hyperparameter tuning process in the transfer learning framework.

i. *Activation and dense layers* We improved model generalization by fine-tuning hyperparameters in our study. To that end, we experimented with adjusting the dense layer of 128 to 4096 units and used ReLU and softmax as activation functions. The goal for this configuration is a suitable equilibrium between the expressiveness of the model and the avoidance of overfitting.
ii. *Dropout rate* In dropping out, we introduce randomness so neurons can have fewer specific connections and thus rely less on overfitting. This disrupts the model and begins to learn generalizable features to remember training data and not overfit. It makes generalized features through dropout but disrupts co-adaptation to conquer overfitting.
iii. *Learning rate* Step size (a direct result of the learning rate) and its effect on convergence and accuracy is one of our study’s key factors of optimization. We systematically probe various values for the learning rate using grid and random search methods to find the best one, leading to higher model performance.
iv. *Batch size* the batch size determines the amount of training sample handled per iteration, shaping up at least the training speed and stability. Batches of larger sizes will speed up learning but require more memory.
v. *Optimizer and pooling* All models used a hyperparameter configuration tuned with tight precision and used Adam Optimizer and MaxPooling2D. This strategic combination not only serves as an encouragement to have strong generalization but also is a solution to the problems of overfitting, guaranteeing robust performance even across very different datasets.
vi. *Number of epochs* The epoch parameter determines the frequency at which we loop through the training dataset in the model. While many epochs can overfit, not enough epochs can underfit. A validation set can assess the number of epochs at which the model performance is best; therefore, utilizing the validation set is ideal.
vii. *Loss and weights* For our study, we improved model precision by combining sparse categorical cross-entropy, which indicates that the network should learn to make more precise class distinctions. The model is driven by using weight decay toward a more straightforward solution and discouraging large weight values to deter overfitting. The parameter weight decay controls the strength of the penalty term and is fine-tuned to result in the best model performance overall. This approach is motivated by a balance between complexity and simplicity in the model, allowing us to obtain closer to the correct accuracy and generalizability.
viii. *Cross-validation* In our study, hyperparameter tuning benefits from cross-validation—an effective technique assessing a model’s generalization performance. This method prevents overfitting by evaluating the model on unseen data, ensuring robustness and enhancing its adaptability to new information.

### Pseudocode for kidney disease classification based on hyper parameter tuned transfer learning mechanism

To achieve optimal accuracy in segmenting kidney stones from surrounding tissues, we set our distance transform threshold to 0.7 in our study. Noise and image quality were reduced using Gaussian filtering to enable better, more accurate feature extraction. In addition, CT images were resized through bilinear interpolation to maintain smooth transitions and preserve important CT image details. Together, these choices increase the model’s ability to classify kidney conditions accurately and, therefore, more often, results in more reliable diagnoses and improved patient outcomes.
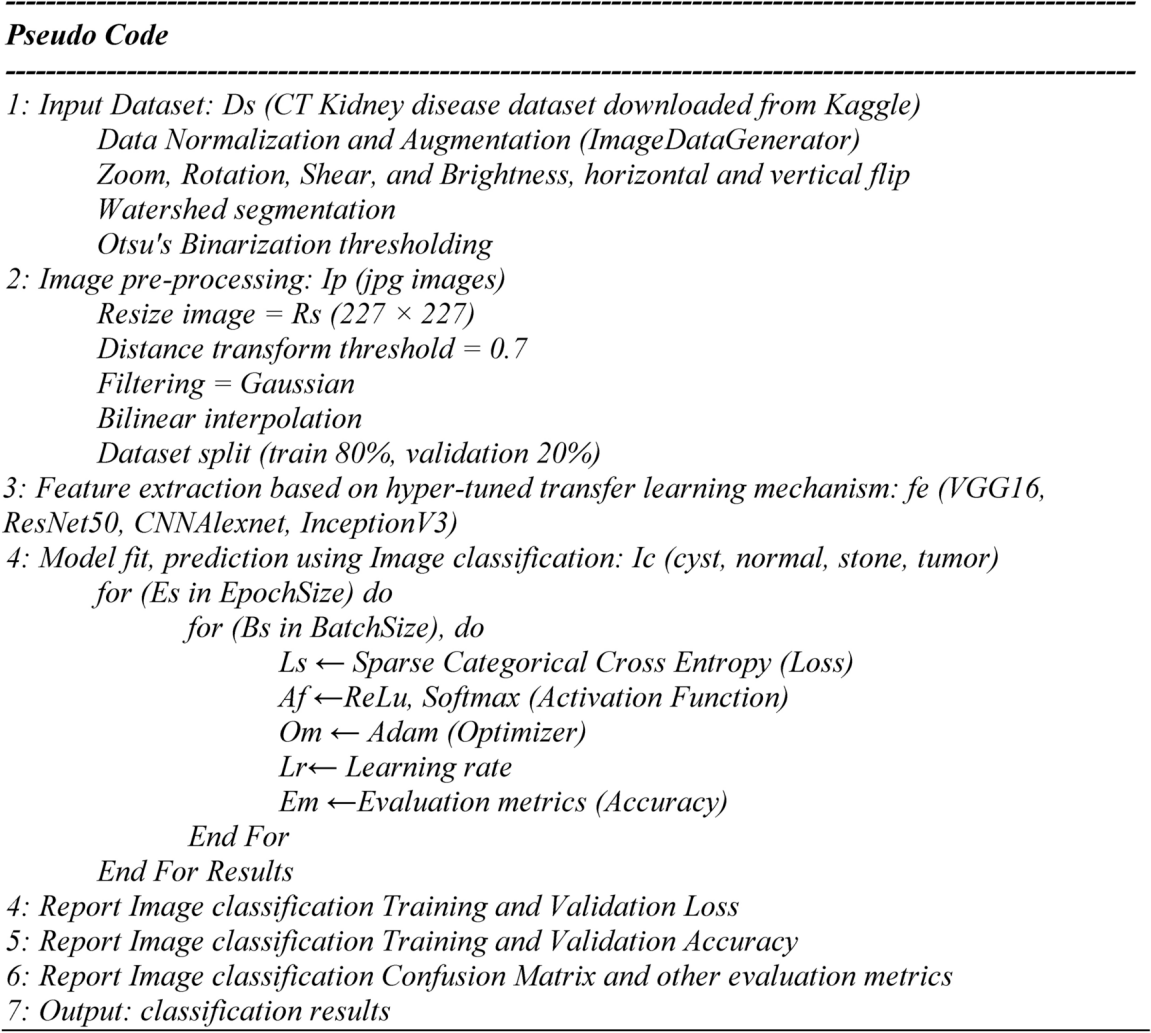


### Experiments setup

In all experiments, fixed parameters to the pre-trained deep models were used, so the same testing environment was used. The experiments were carried out on a computer operating Windows 10 with an Intel i5 processor, 8 GB RAM, and a 500 GB disk. Implementations of the proposed models were executed on the Kaggle platform by exploiting the capacities of the Python language libraries like TensorFlow, Keras, SKlearn, Optimizer, and Backend. Image processing and augmentation were done using Python packages such as OpenCV2. We performed all experiments on a dedicated workstation running on Google Compute Engine with a Kernel-Tesla P100 GPU. Accelerations from computations were enabled with a GPU beaming, Tesla P100, 3584 CUDA cores, and 16 GB GDDR6 VRAM. In combination, these advances in the tools and hardware made our kidney disease classification model much more efficient and effective. It was a single core hyper threaded Xeon processor @2.2 GHz with 15.26 GB available RAM and 155 GB available disk space on the workstation for seamless experimentation.

## Results

This section provides insights into the experiments conducted with five distinct models. Additionally, the hybrid model CNNAlexnet, the VGG16, ResNet50, and the modified CNN, InceptionV3, are used for comparison. Each model has its architecture given shared hyperparameters. The results of these experiments are shown in the subsequent subsections, and the performance of each model is discussed. We evaluate them one by one, through meticulous examination, to determine their distinct capabilities and achieve a complete understanding of their strengths and weaknesses. Such diverse models enhance our scope of kidney disease detection and provide beneficial insights into the adaptation and effectiveness of different neural network ‘architectures’ in image analysis.

Observing the data presented in Tables 4, 5, 6, 7 and 8, it becomes evident that all the fine-tuned models exhibit the highest accuracy among the entire array of models, boasting an impressive 99.96% for the InceptionV3 model and 100% for the VGG16 model. This remarkable performance underscores the efficacy of transfer learning architectures, which power pre-trained weights and knowledge gleaned from extensive datasets. The inherent capability of these models to discern intricate patterns within the kidney tumor dataset substantiates their robustness, enabling accurate predictions and effective differentiation between tumor and normal kidney instances. The upward trend in test and validation accuracies with increased epochs is noteworthy, excluding the initial epochs (1–5). The decision to halt training at 25 epochs stems from the observation that validation accuracies plateau beyond this point, particularly evident in the stable values between epochs 20–25. The efficiency of the fine-tuned CNN model is evident in the relatively short time required for each step, approximately 14 ms/step, while for fine-tuned CNNAlexNet and InceptionV3 models is around 15 ms/step. Enhancing the neural network’s architecture and hyperparameters can significantly improve model performance, reducing the time required for each step and increasing overall accuracy. This strategic approach of optimizing these elements is to optimize processing and obtain better diagnostic results in the kidney disease classification model. Instead of including these improvements in the model at once, we can focus on these to ensure the model works well and meets the needs of the clinical application in kidney disease detection. This paper is of particular interest because it proposes strategic ways to fine-tune these aspects to improve the proposed model’s performance and efficiency in kidney tumor detection and classification.

**Table 4:** Performance metrics for the CNN model.

Epoch	Test accuracy (%)	Validation accuracy (%)	The absolute variance between test and validation accuracy	Time required for each step (ms/step)
1	66.53	76.46	9.93	12
5	79.23	78.95	0.28	9
10	76.08	76.94	0.86	9
15	58.83	65.77	6.94	9
20	69.35	64.28	5.07	9
25	60.89	58.34	2.55	9

**Table 5:** Performance metrics for fine-tuned VGG16 model.

Epoch	Test accuracy (%)	Validation accuracy (%)	The absolute variance between test and validation accuracy	Time required for each step (ms/step)
1	91.91	75.49	16.42	36
5	97.88	96.79	1.09	25
10	99.45	99.96	0.51	25
15	99.89	100.00	0.11	25
20	99.82	99.96	0.14	25
25	99.83	100.00	0.17	25

**Table 6:** Performance metrics for fine-tuned ResNet50 model.

Epoch	Test accuracy (%)	Validation accuracy (%)	The absolute variance between test and validation accuracy	Time required for each step (ms/step)
1	89.82	99.80	9.98	32
5	99.55	99.72	0.17	24
10	99.49	99.96	0.47	24
15	99.59	99.84	0.25	24
20	99.49	99.88	0.39	24
25	99.85	99.92	0.07	24

**Table 7:** Performance metrics for fine-tuned CNNAlexnet model.

Epoch	Test accuracy (%)	Validation accuracy (%)	The absolute variance between test and validation accuracy	Time required for each step (ms/step)
1	69.41	73.44	4.03	43
5	98.57	96.42	2.15	14
10	99.69	94.13	5.56	14
15	99.82	99.96	0.14	14
20	100	99.88	0.12	14
25	100	99.88	0.12	14

**Table 8:** Performance metrics for the fine-tuned InceptionV3 model.

Epoch	Test accuracy (%)	Validation accuracy (%)	The absolute variance between test and validation accuracy	Time required for each step (ms/step)
1	87.22	97.67	10.45	23
5	99.14	99.60	0.46	15
10	99.14	99.60	0.46	15
15	99.93	100.00	0.07	15
20	97.72	99.24	1.52	15
25	99.39	99.96	0.57	15

**Fig. 6:**
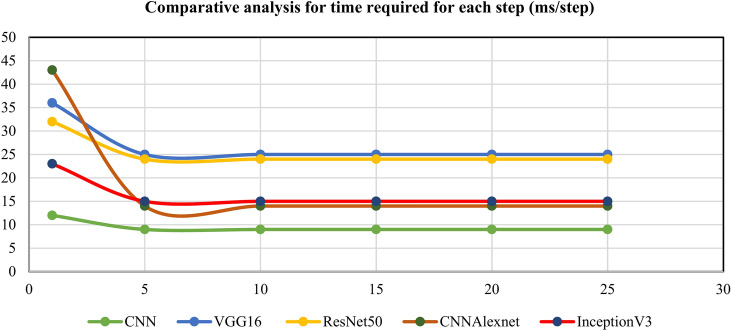
Comparative analysis for time required for each step (ms/step).

**Fig. 7:**
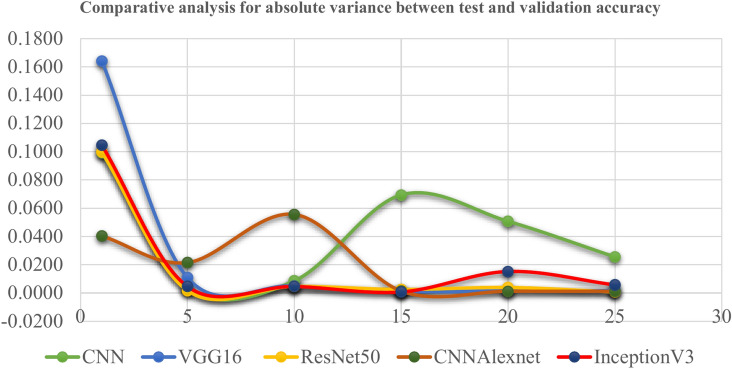
Comparative analysis for absolute variance between test and validation accuracy.

Tables 4, 5, 6, 7 and 8, and we look towards Fig. 6 for a clearer view of the time needed for each step of the kidney disease classification process. The results show that the CNN model is the fastest due to its simpler architecture. However, VGG16 and ResNet50 need more time because they are much more complex. Using hyperparameter tuning, InceptionV3 and CNNAlexnet give us an average step time of around 15 ms, showing their transfer learning robustness. The diagnostic performance Additionally, this analysis highlights the importance of model selection to find a balance between computational efficiency and diagnostic performance. Furthermore, Fig. 7 pulls us toward a comparative study on the absolute variance between test and validation accuracy. The comparative analysis of the accuracy of test and validation also indicates improperly low variance, especially in early epochs. The hyperparameter-tuned transfer learning models converged rapidly after approximately 10–15 epochs. This trend illustrates the robustness and the generalization capability of these models and suggests the potential tendency of the models to overfit or underfit. We analyze these metrics and can use them to identify performance and computational statistics. It will help us later with further refinements and optimizations.

**Fig. 8:**
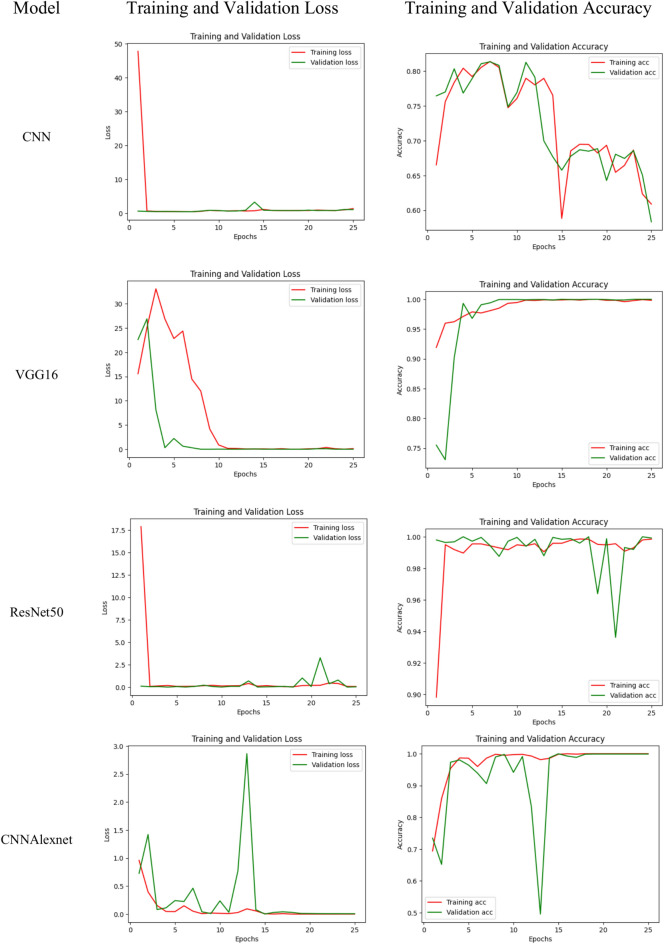

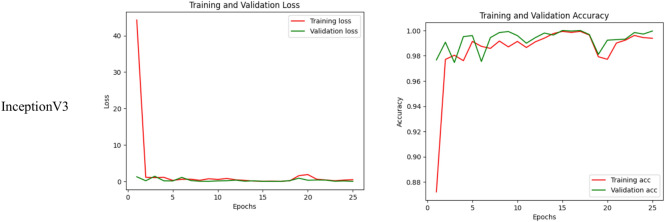
Training, validation loss and training, validation accuracy.

In Fig. 8, you can see the panorama of popular deep learning and transfer learning models that have competed based on the training and validation accuracy of diagnosis in kidney disease. Specific layers may benefit from fine-tuning, and InceptionV3, in particular, appears to experience an increase in the validation accuracy beyond that of ResNet50 trained accuracy. Its transformative potential is underscored by the fact that, just like any good transfer learning, one can adapt pre-trained knowledge to the specific context of kidney disease. The result is improved diagnostic accuracy, with the model’s performance tailored to handle the special issues relevant to kidney disease classification more effectively. Using established models can improve the ability to detect and manage kidney conditions and, hopefully, improve patient outcomes. The figure shows that the optimal model choice is a function of data size and computational resources. Visual representation translates the raw training accuracy to something more than just highlighting adaptability and the influence of transfer learning toward precision medicine. Continued development of these models can generate a revolution in the diagnosis of kidney disease, bringing about the sighting and appropriate treatment of kidney disease at earlier stages.

A key aspect in the design of the proposed framework is the choice of the loss function, which affects its effectiveness and efficiency. This research uses SCCE, which is well-suited for multi-class classification problems. The training and validation loss computations for the various transfer learning models utilized in research can be seen in Fig. 8. The divergence between predicted probabilities and actual class labels is calculated by SCCE, which makes the model aware of its predictions’ effectiveness in assessing categories like cyst, normal, stone, and tumor in kidney disease classification. From a computational complexity point of view, the loss function helps increase the global cost of backpropagation, as it affects the gradient computations during optimization. Since SCCE is approximately O(nC), i.e. SCCE has a logarithmic operation on predicted probabilities, where 'n' is assumed to be the number of samples, and 'C' is the number of classes. Adding this loss function with the Adam optimizer helps minimize computational overhead and accelerates the convergence speed. It also affects the performance of the extraction and classification of features, depending on the function of loss. A correctly optimized loss function helps the model to learn representative things. Nevertheless, we highlight that improper tuning or lack of data augmentation for class imbalances can generate vanishing gradients or biased predictions, to which one must possibly go for data augmentation, class weighting, or focal loss adjustments to achieve robustness.

The confusion matrix is a handy tool for assessing how good a deep learning-based image classification model is. It details statistics for each class in the database – giving true positives, false positives, and false negatives. It gives insight into the model’s good and bad points in recognizing disease classes. This becomes very useful when there is more than one class, as it helps you analyze the model performance across the classes. It identifies classes the model appears to be having difficulty distinguishing to help understand where focused training improvements may be required to overcome this difficulty.

**Fig. 9:**
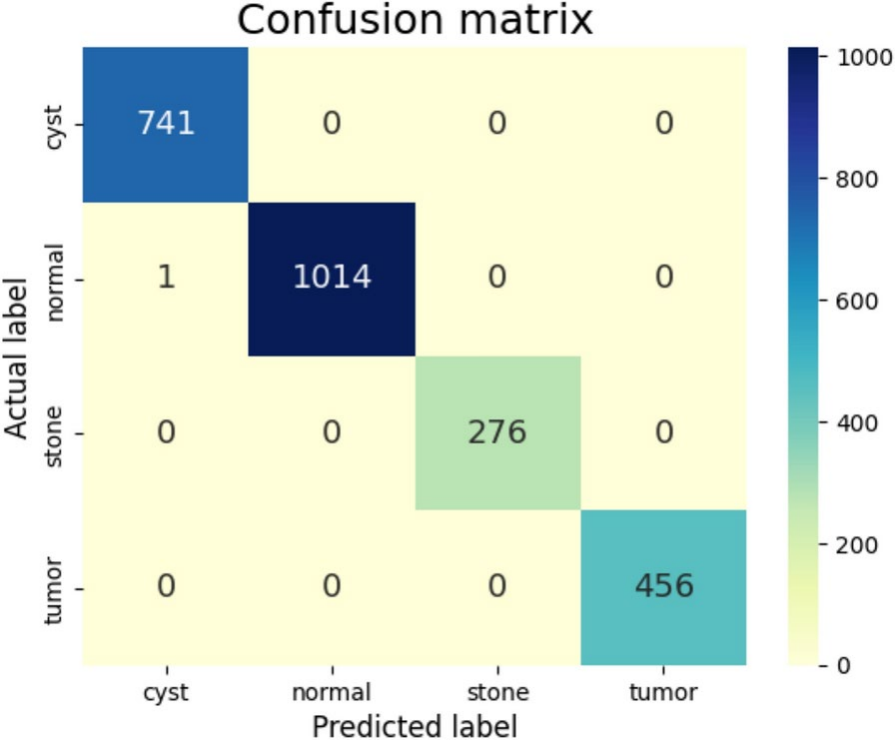
Confusion matrix for fine-tuned transfer learning InceptionV3 model.

In Fig. 9, a confusion matrix illustrates a more detailed version of the fine-tuned transfer learning InceptionV3 model outcomes. The model managed to classify different categories, as shown in the matrix. These results demonstrate that the model is robust and generalizable and is a promising solution for accurately diagnosing kidney disease. The model’s validation accuracies are exceptionally high across diverse classes, reflecting the model’s ability to learn and capture the intricate patterns of each pathology well. Thus, the proposed model not only outperforms its competitors but enables us to claim that it is likely to significantly contribute to medical image analysis, particularly to the early detection and classification of kidney diseases.

Table 9 summarizes the classification performance of several models for classifying kidney diseases on their precision, recall, F1-score, and ROC AUC scores.

**Table 9:** Comprehensive performance evaluation of implemented models using key metrics.

Model/metrics	Precision	Recall	F1-Score	ROC-AUC
CNN	36.83	40.22	58.34	64.93
VGG16	90.96	88.08	91.40	94.16
ResNet50	99.95	99.90	99.92	99.93
CNNAlexnet	99.90	99.79	99.88	99.92
InceptionV3	99.98	99.90	99.96	99.97

The results show that the proposed hyperparameter tuning and transfer learning approaches improve model performance considerably better than the currently known solutions. Interestingly, metrics such as precision and recall from ResNet50, CNNAlexnet, and InceptionV3 were close to 1 (99%) or higher. This proves they are robust in correctly detecting disease conditions like cysts, stones, and tumors in the kidney. Hyperparameter optimization integrated into the model architectures was used to fine-tune them and achieve maximum performance, and transfer learning was used to utilize previously learned features of pre-trained models. Thus, these strategies significantly enhanced diagnostic accuracy and efficiency in classifying CT images of kidney diseases. Optimization of these elements on the models resulted in increased pattern recognition capability, which, in turn, enhanced the reliability and accuracy of diagnosis in clinical settings. The work of our approach is that these advancements put it forward as a first-class advanced arrangement in kidney infection location, as it surpasses typical techniques by a long shot and conveys better results to patients (given quicker and precise diagnoses).

The results of Table 10 show the powerful ability of fine-tuned state-of-the-art transfer learning models: VGG16, ResNet50, CNNAlexnet, and InceptionV3 to achieve high accuracies, outperforming other methods. Results with substantial increases in model accuracies paint a picture of the benefits of using transfer learning and hyperparameter fine-tuning. We also performed ablation studies on the effect of different image processing techniques (data augmentation, for example, rotation, zoom, and shear) on model performance. It turned out that these techniques combined resulted in a substantial overfitting reduction and a better generalization ability of the model. More noteworthy, InceptionV3 achieved a distinguished accuracy of 99.96 percent in the classification task, which particularly distinguishes them from other competitors. Seeing such excellent performance of fine-tuned transfer learning models stresses the importance of exploring other models and methodologies for better results in image classification. In addition to the excellence of particular models, these results indicate the need for continual exploration and comparison of approaches. Finely tuned transfer learning models achieve outstanding accuracy, which strongly inspires investigation into their potential for broader applications, suggesting there are dynamics to the advancements towards excellence through methodology.

**Table 10:** Performance comparison with other state-of-the-art methods.

References	Model	ACC	EPOCH	Image Pixel Size
Subedi et al.^16^	Resnet50	73.80	20	224 × 224
Pande and Agarwal^14^	YOLOv8n-cls	82.52	50	128 × 128
Zhang et al.^28^	Cascading RCNN, ResNet101	87.10	45	512 × 512
Pal^45^	Ensemble SVM	93.00	-	-
Parakh et al.^39^	Cascading CNN	95.40	-	512 × 512
Ugur Kilic et al.^17^	YOLOv4	96.10	60	416 × 416
Muneer Majid^42^	ResNet-101	96.67	35	128 × 128
Yildirim et al.^46^	XResnet-50	96.82	40	224 × 224
Sohaib Asif et al.^43^	InceptionV3	97.24	-	224 × 224
Ahmed et al.^41^	Modified VGG16 model	97.41	10	224 × 224
Sohaib Asif et al.^43^	InceptionResNetV2	97.64	-	224 × 224
Dalia Alzu’bi et al.^37^	2D-CNN	97.70	50	224 × 224
Li et al.^20,21^	Transfer Learning based Dual-Augmentation	97.99	-	–
Subedi et al.^16^	VGG16	98.20	20	224 × 224
Muneer Majid^42^	DenseNet-121	98.22	35	128 × 128
Sohaib Asif et al.^43^	StackedEnsembleNet	98.43	-	224 × 224
Sohaib Asif et al.^43^	PSOWeightedAvgNet	98.43	-	224 × 224
Subedi et al.^16^	Vision Transformer	99.64	20	224 × 224
This Research (2024)	Fine-tuned InceptionV3	99.96	25	227 × 227

Floating-point operations (FLOPs) and the number of arithmetic operations are applied to floating-point numbers. Matrix multiplications, activations, and gradient calculations are all basic operations in machine learning; these are the ones these operations act upon. FLOPs are a critical metric for quantifying a model’s computational cost or complexity, which could be the heuristic of the total arithmetic operations. Such measurement is necessary for evaluating computation efficiency and the model performance. Different processing stages of the proposed framework have different computational complexities, as shown in Table 11. Watershed segmentation is O(n log n) as the approach uses a marker-based sorting, while Otsu’s thresholding is O(n) as it analyzes pixel intensity in O(n). Gaussian filtering and bilinear interpolation are pixel-wise transformations with an O(n^2^) complexity. During the training phase, several factors relevant to computational cost are epoch (E), batch (B), feed-forward or feed-backward network (F), kernel size (k), channel size (C), image resolution (H × W), and total number of images (n). The most computationally demanding step in all steps is feature extraction, which performs O(n^3^), as it needs to involve many convolutions across layers. The influence of this step on the overall efficiency of the framework is such that computational optimization is necessary for large-scale datasets and real-time applications^47–49^. GPU parallelization significantly reduces it by sharing the computational load and optimizing performance. This helps in higher processing speed and good performance of the model in kidney disease classification. Motivated by deep learning optimizations and in the context of high-performance computing, it is proposed to use deep learning to obtain an accuracy-efficiency balance when dealing with large-scale medical imaging datasets. Dense layers make AlexNet and VGG16 have high computational demand, but ResNet50 does so with the residual connection. Factorized convolutions and parallel processing are what InceptionV3 optimizes performance with. InceptionV3 has the best balance of accuracy and efficiency for real-time applications. For more complicated networks, e.g. ResNet50, it is preferable, while VGG16 and AlexNet are rigid to an environment with low resources, as their computation is more costly.

**Table 11:** The computational complexity for state-of-the-art methods.

Model	Layers	Parameters	Memory (MB)	FLOPs (Billion)	Complexity	Key Observation
AlexNet	8	60 M	230 MB	1.5B	O(n^2^)	Simplicity, an early breakthrough in deep learning
InceptionV3	42	23 M	92 MB	5.0B	O(n log n)	Optimized architecture, best efficiency vs. accuracy trade-off
ResNet50	50	25 M	98 MB	3.8B	O(n^2^ log n)	Residual learning reduced vanishing gradients
VGG16	16	138 M	528 MB	15.5B	O(n^3^)	High accuracy but heavy computational burden

## Conclusion and future scope

This research devised an all-encompassing strategy to classify CT scan images, explicitly focusing on distinguishing between four clinically significant categories: Normal, Cyst, Tumor, and Stone. The aim was to improve the efficiency of dealing with medical image data using modified transfer learning methods and hyperparameter tuning models. Morphological patterns are then quantified by four fine-tuned transfer learning models, VGG16, ResNet50, CNNAlexnet, and InceptionV3, and introduced into the framework for predicting kidney tumors. The models were thoroughly evaluated through various metrics and compared to existing approaches. Finally, test results showed the effectiveness of fine-tuned transfer learning models, with an impressive 99.96% accuracy when run on InceptionV3. This has shown their superiority in detecting kidney disease. The results reported in Tables 3, 4, 5, 6, 7, 8, 9 and 10 confirm the effectiveness of the proposed approach. This research significantly contributes to medical image analysis by integrating a new transfer learning and hyperparameter tuning model into the classification pipeline. The success of the innovative approach both embraces and advances the boundaries of traditional methods, paving the way to significant advancements across the domain.

Results from experiments show that advanced techniques for transfer learning can help classify kidney CT scans. The use of generated data resolves the dependence of the methodology on specific instances and delivers strong kidney tumor classification performance. The importance of multi-modal techniques is highlighted by elevated accuracy, and transfer learning and hyperparameter tuning convey progress in kidney disease detection. The approach reliably identifies kidney conditions, including cysts, tumors, and normal ones; these current findings support it. Future work could develop other methodologies, such as multi-dataset evaluation, to validate the model’s generalizability to different demographics and imaging conditions. Therefore, advanced imaging techniques can be integrated with new algorithms, including the chi-square and the firefly optimization, to enhance classification accuracy. Some strategies for dealing with class imbalance (including oversampling and creating synthetic data (e.g., SMOTE)) can also be studied. To achieve model interpretability while addressing clinical data alongside imaging result integration, explainable AI techniques will be implemented to enhance his interpretability. It may integrate the IoT-based kidney stone detection module with healthcare and telemedicine applications in future work research. We also explore real-time deployment in clinical workflows and adversary robustness. Out of the box, the model can be used as an ongoing research tool, and extraction, optimization, and ensemble deep learning models, as well as other transfer learning models, can be used to try to improve the model’s performance. Further research could expand the model generalization using a more extensive and diverse kidney CT dataset. Moreover, integrating multi-modal imaging modalities, including MRI and ultrasound, would also add to the diagnostic accuracy, robustness and clinical applicability^50^.

## Data Availability

The datasets used in this study are publicly available and can be accessed through the below-mentioned links. https://www.kaggle.com/datasets/nazmul0087/ct-kidney-dataset-normal-cyst-tumor-and-stone?select=kidneyData.csv
